# Hormone refractory prostate cancer: Current understanding and future perspectives

**DOI:** 10.4103/0970-1591.30264

**Published:** 2007

**Authors:** Makarand Khochikar

**Affiliations:** Department of Uro-oncology, Siddhi Vinayak Ganapati Cancer Hospital, Miraj - 416 410, India. E-mail: khochikar@rocketmail.com

Despite the ‘PSA era’ a large number of prostate cancer patients in India have locally advanced /metastatic disease at presentation. This is a uniform scene across the length and breadth of our country. Things have started changing slowly in the metros and areas where superspecialty services are in practice, but it would be a while till we reach a stage where we succeed in catching them at an early stage. The overall outcome of metastatic prostate cancer is dismal. However, with the advent of better understanding of molecular biology, androgen receptors newer interest is generated in improving the outcomes of the treatment in metastatic prostate cancer. This symposium is aimed at addressing some of the key issues in the current understanding of hormone refractory prostate cancer (HRPC) and the various treatment options we have and would have in future.

Is the term ‘;hormone refractory prostate cancer’ synonymous with ‘androgen independent/insensitive prostate cancer’? They are not the same. The newer definitions have enabled us to understand the difference in these two conditions. We would be able to learn the difference between these two terminologies in this symposium.

We all know that prostate cancer is androgen-sensitive in the initial stage and depends on the androgen receptor (AR) to mediate the effects of androgens. However, the exact mechanism of HRPC is not yet completely understood. The article Molecular biology in prostate cancer and HRPC reviews the current literature and understanding in this respect. Numerous potential mechanisms like AR amplification, over-expression or mutation or alterations in AR signaling pathways are not only important in understanding the basic mechanism in HRPC but would also enable us to have future treatment strategies.

There are many factors and parameters that decide the fate of a patient of metastatic prostate cancer. Apart from clinical parameters and PSA dynamics, predictive nomograms and tables have become an important tool in counseling the patients and planning the treatment. Use of nomograms and statistical models are the key words in modern practice and I guess they are going to stay. What lies in the future of these patients is hard to predict, but the use of such models would take us a long way in being practical in treating these patients.

There is a large spectrum of clinical problems we face in HRPC. Its mechanism, clinical presentation and appropriate management are important aspects of treatment of HRPC. These issues are covered in depth in the clinical problems and their management in this symposium.

Use of biphosphonates in metastatic breast cancer and multiple myeloma is an established standard of care. Its usefulness in prostate cancer has been debated since the last decade. An apt review on this issue will enable us to select the patients who would benefit most with biphosphonates and the controversial issue of assessing bone marrow density is also addressed.

Palliative or supportive care vs. cancer-specific therapy is yet another emerging issue in the management of HRPC. Palliative /supportive care is no doubt very important, but in the light of some success we have achieved in taxane-based chemotherapy, the issue of cancer chemotherapy in prostate cancer is the buzzword. The article on chemotherapy takes an in-depth review of the clinical trials so far in chemotherapy, the success we have achieved so far and the unresolved issues and future of chemotherapy. It's amazing to see the entire concept getting changed from the so-called chemo resistant nature of the prostate cancer to ‘chemo-sensitive’ now.

So, what lies in the future of treatment of HRPC? Is it immunotherapy; is it gene therapy or anything different? The article on newer therapeutic targets has covered in detail the newer approaches, with great simplicity.

An attempt has also been made to draw a treatment algorithm for HRPC patients for practical use. There are many ways of treating the problems in HRPC like ‘horses for the courses’ but one would use one's own judgment in those.

I sincerely hope the readers find this symposium interesting and useful in managing their patients with HRPC.

